# Low Serum and Urine Fetuin-A Levels and High Composite Dietary Antioxidant Index as Risk Factors for Kidney Stone Formation

**DOI:** 10.3390/jcm14051487

**Published:** 2025-02-23

**Authors:** Mehmet Arif Icer, Tevfik Koçak, Yusuf Icer, Emine Kocyigit, Duygu Ağagündüz, Makbule Gezmen-Karadag, Suleyman Yesil, Ferenc Budán

**Affiliations:** 1 Department of Nutrition and Dietetics, Faculty of Health Sciences, Amasya University, 05100 Amasya, Turkey; 2 Department of Nutrition and Dietetics, Faculty of Health Sciences, Gümüşhane University, 29100 Gümüşhane, Turkey; tkocak@gumushane.edu.tr; 3 Centre for Clinical Brain Sciences, The University of Edinburgh, Edinburgh EH16 4SB, UK; yusuf.icer@ed.ac.uk; 4 Department of Nutrition and Dietetics, Faculty of Health Sciences, Ordu University, 52200 Ordu, Turkey; kocyigitem@gmail.com; 5 Department of Nutrition and Dietetics, Faculty of Health Sciences, Gazi University, 06490 Ankara, Turkey; duyguturkozu@gazi.edu.tr (D.A.); mgezmen@gazi.edu.tr (M.G.-K.); 6 Department of Urology, School of Medicine, Gazi University, 06490 Ankara, Turkey; syesil2003@yahoo.com; 7 Institute of Physiology, Medical School, University of Pécs, H-7624 Pécs, Hungary

**Keywords:** fetuin-A, nephrolithiasis, composite dietary antioxidant index, dietary intake

## Abstract

**Background:** Fetuin-A prevents the precipitation of hydroxyapatite in supersaturated solutions of calcium and phosphate; however, its relationship with nephrolithiasis has yet to be clarified. The aim of this study was to investigate the protective and predictive roles of serum and urine fetuin-A levels in nephrolithiasis and their relationships with the composite dietary antioxidant index (CDAI). **Methods:** This study involved 75 adult patients with kidney stone disease and 71 healthy adults without kidney stone disease in the control group. Participants had specific anthropometric measurements taken, and three-day food records were kept. The CDAI was calculated by summing six standard antioxidants, including vitamins A, C, and E, manganese, selenium, and zinc, representing participants’ antioxidant profile. In addition to some analyzed serum and urine parameters of the participants, fetuin-A levels were measured using the enzyme-linked immunosorbent assay (ELISA) method. **Results:** In patients with kidney stones, both serum and urine fetuin-A levels (676.3 ± 160.14 ng/mL; 166.6 ± 128.13 ng/mL, respectively) were lower than in the control group (1455.6 ± 420.52 ng/mL; 2267.5 ± 1536.78 ng/mL, respectively) (*p* < 0.00001). In contrast, the CDAI was higher in patients with kidney stones compared to those without kidney stones (*p* < 0.001). Besides, several dietary parameters had significant positive correlations with serum and/or urinary fetuin-A. **Conclusions:** The present study suggests that serum and urinary fetuin-A levels may serve as protective factors against kidney stones and could potentially be used as predictive markers for the development of nephrolithiasis. Furthermore, our results suggest that the CDAI above a certain level may increase the risk of stone formation and that some dietary parameters may affect the levels of this biomarker in serum and urine.

## 1. Introduction

Kidney stones, also called nephrolithiasis or urolithiasis, are hard mineral (calcium, oxalate, and phosphate) deposits formed from minerals dissolved in urine and are typically excreted through the urethra [1,2]. The prevalence of urolithiasis has increased significantly from past to present [3], and the recurrence rate is estimated to be approximately 50% in 10 years. Kidney stone disease has a multifactorial etiology consisting of environmental, dietary, hormonal, and genetic components [4].

One common ectopic calcification that occurs in the kidneys is nephrolithiasis [5]. For this reason, in recent years, there has been increasing focus on calcification inhibitors [6]. Fetuin-A, sometimes referred to as alpha-2-Heremans-Schmid glycoprotein, is a systemically potent inhibitor of ectopic calcification and is one of the main inhibitory factors being studied [7]. To prevent calcification, fetuin-A can handle excess calcium and phosphate loads by forming a soluble and highly stable fetuin-mineral complex (FMC) or calciprotein particles (CPP) [8]. Fetuin-A has been shown to prevent the precipitation of hydroxyapatite from a supersaturated solution of calcium and phosphorus in vitro [6]. Because of its strong affinity for binding calcium/phosphate, fetuin-A has also been shown to inhibit the formation and deposition of calcium phosphate crystals [9,10]. Apart from these potential processes, it is also believed to prevent kidney stones by lowering inflammation, which leads to oxidative stress [11]. Patients with urolithiasis have lower serum and urine fetuin-A levels than controls, according to some earlier research [6,12]. However, there is a study in the literature that showed no significant difference in serum and urine fetuin-A levels between groups with and without urolithiasis [13].

Dietary changes can prevent kidney stones and reduce invasive surgeries. Identifying risk factors helps plan dietary protocols. High protein intake increases acid load, while antioxidant compounds reduce oxidative stress and calcium oxalate crystal formation, thus reducing kidney stone formation [14,15]. Based on the consumption of different dietary antioxidants, such as vitamins A, C, and E, manganese, selenium, and zinc, the Composite Dietary Antioxidant Index (CDAI) is a tool used to evaluate the general antioxidant capacity of a person’s diet [16]. Higher CDAI scores are linked to a lower risk of kidney stones, according to Huang et al. [17] However, it has also been suggested that both lower and higher CDAI scores may be associated with a higher prevalence of kidney stones [18]. The relationship between this index and kidney stones is still unclear. Determining dietary factors affecting serum and urinary fetuin-A levels can clarify the link between kidney stone formation and potential dietary variables that could modulate these levels [19].

The role of fetuin-A in the etiology of nephrolithiasis is not yet clear and the results are contradictory [6,12,13,20]. A limited number of studies have evaluated serum and urine fetuin-A levels together in kidney stone patients [6,8,12,13]; however, dietary parameters and dietary antioxidant index should also be evaluated together. In addition, the relationship between the CDAI and low serum and urine fetuin-A levels, which is considered as one of the possible risk factors for the development of kidney stones, has not been investigated yet. This study was conducted to investigate the relationships between serum/urinary fetuin-A levels, certain dietary parameters, and the CDAI in relation to the risk of kidney stone formation, evaluate the effects of nutrition on fetuin-A levels, and find an independent predictive factor for kidney stone development.

## 2. Materials and Methods

### 2.1. Study Design and Participants

**Figure 1:**
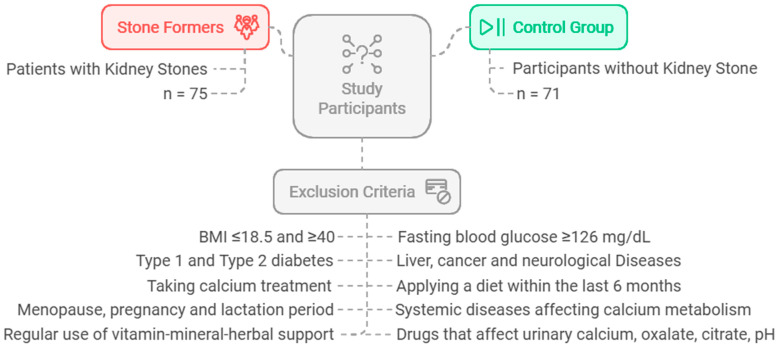
The flowchart and exclusion criteria of the study.

The sample size was estimated assuming a 5% alpha error (i.e., the confidence level = 95%) and 80% power. The minimal sample size for each group was 65 participants, based on the difference among independent means. The software used for sample size calculation was G*Power 3.0.10. This investigation was carried out on a total of 146 individuals: 75 patients (aged 18–65) who were diagnosed with active kidney stone disease by doctors at the Gazi University Medical Faculty Research and Practice Hospital Urology Department, Turkey, and 71 healthy participants without kidney stone disease. The control group consisted of healthy individuals who were matched to the patient group in terms of age, body mass index (BMI), and gender, and who had no clinical complaints or symptoms supporting kidney stone disease. The flowchart and exclusion criteria for the study are shown in Figure 1.

Researchers used a face-to-face interviewing technique to ask participants about their age, gender, marital status, education level, alcohol consumption status, kidney stone recurrence status, number of main meals and snacks, and family history of kidney stones.

Ethics committee approval was obtained from the Gazi University Faculty of Medicine Clinical Research Ethics Committee (decision number 412, 30 May 2022). The study was conducted in accordance with the principles of the Declaration of Helsinki, and informed consent was obtained from all participants.

### 2.2. Biochemical Analyses

Following at least eight hours of fasting the previous night, blood samples and spot urine samples were collected from each participant in the morning. Serum samples were analyzed for fetuin-A (ng/mL), potassium (mg/dL), phosphorus (mg/dL), uric acid (mg/dL), and creatinine (mg/dL). Urine samples were analyzed for fetuin-A (ng/mL), oxalate (mg/dL), citrate (mg/dL), calcium (mg/dL), potassium (mEq/L), magnesium (mmol/L), creatinine (µmol/L), and uric acid (mg/dL). The biochemistry lab at Gazi University’s Faculty of Medicine in Turkey conducted these analyses.

*Analysis of serum and uriner fetuin-A:* After 15 min of centrifuging the blood samples at 3000 rpm, the sera were transferred into 1.5 mL Eppendorf tubes. Urine samples were centrifuged at 3000 rpm in 10–15 mL conical tubes for 10 min and then placed in 1.5 mL Eppendorf tubes. Prior to analysis, these samples were kept at −80 °C. Serum and urinary levels of Fetuin-A were measured using a special kit (Human Fetuin-A ELISA Kit/Elabscience Biotechnology Inc. Houston, Texas, USA) and the enzyme-linked immunosorbent assay (ELISA) method.

### 2.3. Anthropometric Measurements

Participants’ body weight (kg), body fat percentage, body muscle mass (kg), and body water percentage were analyzed using the Tanita BC 601 bioelectrical impedance device. The working principle of the device is based on the difference in electrical current permeability of lean tissue mass and fat. Impedance was measured against the weak electrical current (50 kHz) applied during procedure. Participants were told that in order to take the measurement accurately, they needed to fast for at least four hours prior to the measurement, avoid drinking water, tea, or coffee, avoid urinating, avoid strenuous physical activity for twenty-four hours prior, and avoid having any metal objects touch their skin while taking the measurement.

Height was measured using a portable Leicester brand stadiometer with the heads in the Frankford plane and participants taking deep breaths. Participants’ waist and hip circumferences were measured by researchers using a non-stretchable plastic tape measure. BMI of participants was calculated with the equation “body weight/height^2^ (kg/m^2^)”. The obtained BMI values were classified based on the WHO classification: 18.5–24.9 kg/m^2^ as normal weight, 25.0–29.9 kg/m^2^ as overweight, and ≥30 kg/m^2^ as obese [21].

### 2.4. Dietary Assessment

Three-day food records (consecutive days: two weekdays and one weekend day) were taken by the researchers to determine participants’ daily dietary energy and nutrient intakes. “Meal and Food Photograph Catalog” [22] and “Standard Recipes for Caterings” [23] books were used to determine food quantities and the amounts included in the portions of the meals. The data were evaluated using computer-assisted nutritional program called Nutrition Information Systems (BeBiS) [24].

### 2.5. Assessment of Composite Dietary Antioxidant Index (CDAI)

Three-day food records were used to obtain information on dietary antioxidants and various food elements intake. To compute the CDAI, we employed a modified version of the Wright et al. developed CDAI protocol [25]. The antioxidant profile of participants was represented by the CDAI, which was computed by adding up six common antioxidants, such as zinc, manganese, selenium, and vitamins A, C, and E. To put it briefly, each antioxidant’s standardized value was calculated by deducting the mean and dividing by the standard deviation. The standardized values of each of the six antioxidants were then added up to determine the CDAI; a higher score denoted a higher consumption of antioxidants from food. The average of three 24 h recall interviews was used to collect data on dietary antioxidants. To ensure data accuracy and reliability, individuals with unusually high or low total energy intake (>4200 or <800 kcal/day for men; >3500 or <500 kcal/day for women) were excluded from the analysis [26]. The CDAI for each participant was calculated using the following formula:

$$\mathrm{CDAI}=\sum_{i=1}^{n=6}\frac{Individual\;Intake-Mean}{Standard\;Deviation}$$

### 2.6. Statistical Analysis

The differences between category variables were examined using the chi-square test. The Mann–Whitney U test statistics were applied to compare the measurement values between two independent groups in data that do not follow a normal distribution. Statistical comparisons across groups were conducted utilizing a two-tailed Student’s *t*-test to examine mean differences for the box plots and heatmaps. Heatmaps were plotted to provide pairwise comparisons of quartiles (Q1–Q4) for serum and urine fetuin-A levels, with color-coded cells signifying *p*-value ranges (e.g., *p* < 0.05, *p* < 0.01) and arrows denoting the direction of change. The significance and direction of linear correlations between fetuin-A levels (serum or urine) and different dietary parameters were evaluated for the scatter plots using Pearson’s correlation coefficient (r). The importance of the relationships was indicated by the given corresponding *p*-values. The *p* values under 0.05 were evaluated as significant. Seaborn (version 0.13.0), a Python (version 3.9.13) utility, was used to plot the graphs [27].

## 3. Results

Table 1 displays the individuals’ baseline characteristics. The mean age of the kidney stone formers was 42.1 ± 10.62 years, and 68.0% of them were male. The mean age of the control group was 39.3 ± 11.16 years, with approximately half (54.9%) being male. The kidney stones group had a higher family history, waist circumference, and waist/hip ratio compared to the control group but lower alcohol use, snack consumption, and education beyond high school (*p* < 0.05).

**Figure 2:**
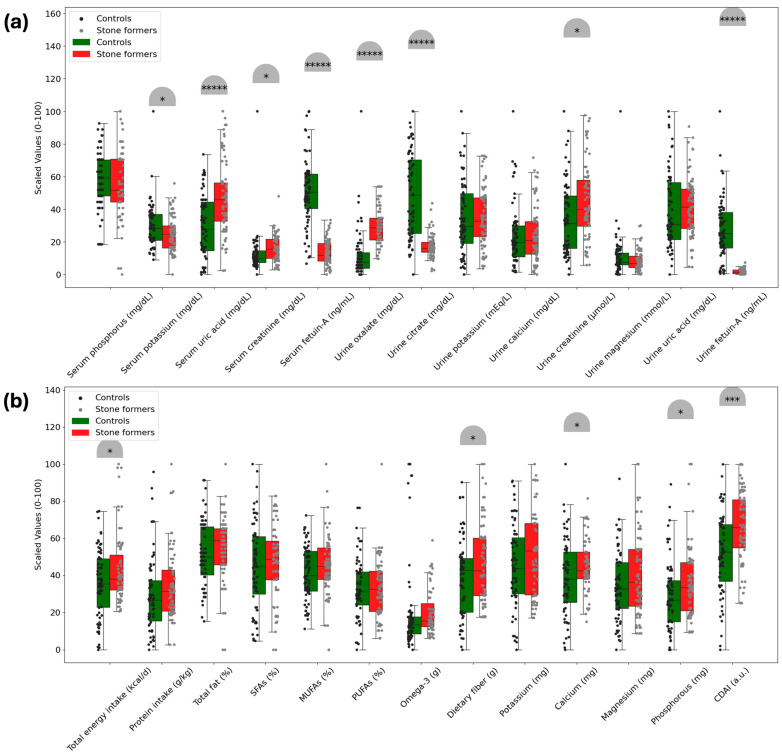
Boxplots demonstrating the differences in nutritional and biochemical parameters between stone formers (red) and controls (green), with scaling them between 0 and 100. (**a**) The top panel illustrates variations in urine and serum biochemical markers, such as urine oxalate, citrate, potassium, and other metabolites, and serum phosphorus, potassium, uric acid, and creatinine. (**b**) Dietary parameters including total energy consumption, fat composition (SFA, MUFA, PUFA), and nutritional levels (calcium, magnesium, phosphorus, etc.) are highlighted in the bottom panel. Signs above the boxplots denote significant differences between groups (* *p* < 0.05, *** *p* < 0.001, ***** *p* < 0.00001). Data variability is displayed by error bars, and distribution is represented by superimposing individual data points.

Based on the analysis of serum biomarkers and dietary parameters, Figure 2 illustrates the significant differences between the control and kidney stone groups. Both serum and urine fetuin-A levels were lower in the kidney stone group (676.3 ± 160.14 ng/mL; 166.6 ± 128.13 ng/mL, respectively) than in the control group (1455.6 ± 420.52 ng/mL; 2267.5 ± 1536.78 ng/mL, respectively) (*p* < 0.00001). Serum potassium and urine citrate levels were also lower in the kidney stone group (*p* < 0.05; *p* < 0.00001, respectively). Serum uric acid, creatinine, and urine oxalate and creatinine levels were higher in the kidney stone group than in the control group (*p* < 0.00001; *p* < 0.05; *p* < 0.00001; *p* < 0.05, respectively).

Participants with kidney stones had higher dietary energy, fiber, calcium, and phosphorus intakes compared to the control group, as observed in a study (*p* < 0.05). Furthermore, the CDAI score was found to be higher in the kidney stone group (2.140286 ± 4.257611 a.u.) than in the control group (−0.765951 ± 4.598879 a.u.) (*p* < 0.05), but no significant differences were observed in other dietary parameters (*p* > 0.05).

**Figure 3:**
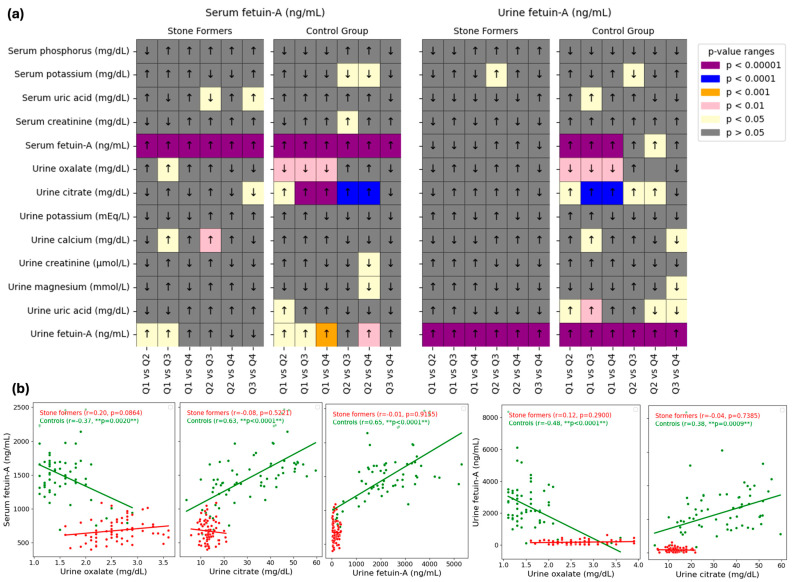
Fetuin-A levels in serum and urine and their relationship to biochemical markers. (**a**) Heatmaps showing the levels of fetuin-A in the serum and urine of stone formers and controls. Dietary parameters were categorized into quartiles (Q1–Q4) based on blood and urine fetuin-A levels in order to evaluate their relationship with dietary parameters. The *p*-value ranges for statistical significance are represented by colored cells, with darker colors denoting more significance (purple for *p* < 0.00001, blue for *p* < 0.0001, orange for *p* < 0.001, pink for 0.01, and yellow for *p* < 0.05). Arrows demonstrate the direction of change between quartiles. (**b**) Scatterplots showing relationship between fetuin-A levels (serum or urine) and urinary parameters in stone formers (red) and controls (green). Regression lines and correlation coefficients (r) with corresponding *p*-values are shown for the groups. Signs above the scatterplots denote significant differences (** *p* < 0.01).

The relationships between fetuin-A (serum and urine, independently) and biochemical markers were examined in Figure 3. The pairwise comparisons of serum and urine fetuin-A levels (ng/mL) for stone formers and control groups over quartiles (Q1 to Q4) are shown in the heatmaps in Panel a. In the assessment of fetuin-A, significant differences were observed between some quartiles based on serum and urinary fetuin-A for different parameters (*p* < 0.05). Most notable differences by quartile of both serum and urinary fetuin-A were noticed for urine oxalate (lower in Q2, 3, and 4 than in Q1) and urine citrate (higher in Q2, 3, and 4 than in Q1; and higher in Q3 and 4 than in Q2). Another most significant difference was observed for urinary fetuin-A by quartile of serum fetuin-A (lower than Q1 in Q2, 3 and 4; and higher than Q2 in Q4).

The relationship between urinary parameters (such as urine oxalate and urine citrate) and fetuin-A levels (serum or urine) is shown by scatter plots in Panel b. Two groups are present in each plot: controls (green points) and stone formers (red points). For every group, linear regression lines are fitted, and the plot legends display the *p*-values and Pearson’s correlation coefficients (r). In the control group, serum and urine fetuin-A correlation with urine oxalate were negative (serum, r = −0.37, *p* = 0.02; urine, r = −0.48, *p* < 0.0001) while their correlation with urine citrate was positive (serum, r = 0.63, *p* <0.0001; urine, r = 0.38, *p* = 0.0029). Additionally, there was a positive correlation between serum and urine fetuin-A levels in the control group (r = 0.65, *p* < 0.0001), with no significant correlation in the stone formers group for all parameters.

**Figure 4:**
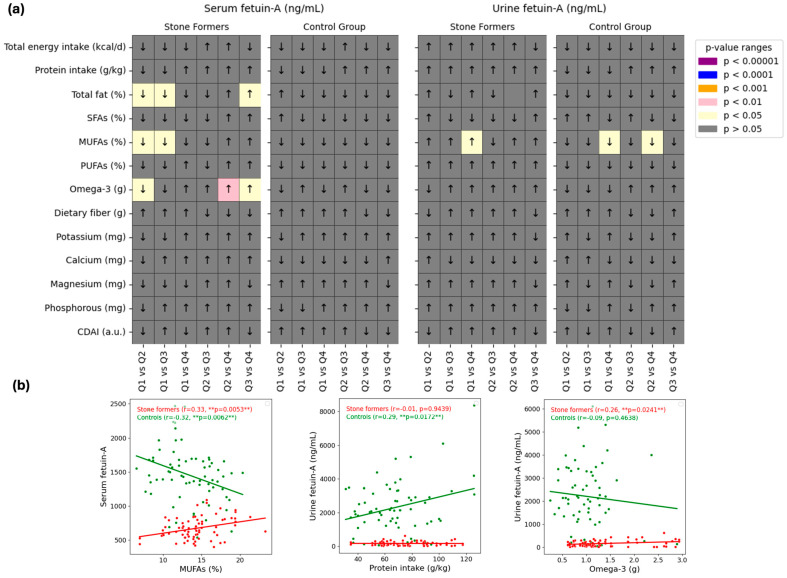
Fetuin-A levels in serum and urine and their relationship to dietary parameters. (**a**) Heatmaps of serum and urine fetuin-A levels in stone formers and controls. To assess their association with dietary parameters, dietary parameters were divided into quartiles (Q1–Q4) according to serum and urine fetuin-A levels. Arrows denote the direction of change between quartiles, with colored cells denoting *p*-value ranges for statistical significance (purple for *p* < 0.00001, blue for *p* < 0.0001, orange for *p* < 0.001, pink for 0.01, and yellow for *p* < 0.05). (**b**) Scatterplots illustrating relationships between several dietary characteristics and fetuin-A levels (serum or urine) in stone formers (red) and controls (green). For each group, regression lines, correlation coefficients (r), and *p*-values are displayed. Signs above the scatterplots denote significant differences (** *p* < 0.01).

Relationships between fetuin-A (serum and urine, independently) and dietary parameters were examined in Figure 4. The pairwise comparisons of serum and urine fetuin-A levels (ng/mL) for stone formers and control groups over quartiles (Q1 to Q4) are shown in the heatmaps in Panel a. In the assessment of serum fetuin-A, significant differences were observed between some quartiles based on serum fetuin-A for dietary total fat (%), MUFAs (%), and omega-3 (g) intakes in the stone former group (*p* < 0.05), with the most notable difference for omega-3, where participants in Q4 had higher intake than those in Q2 and Q3. Significant differences were observed in urine fetuin-A quartiles for dietary MUFAs (%) in both groups (*p* < 0.05).

The relationship between dietary parameters (such as dietary protein and omega-3 intake) and fetuin-A levels (serum or urine) is shown by scatter plots in Panel b. In the kidney stone group, serum fetuin-A was positively correlated with dietary monounsaturated fatty acids (MUFAs) intake (r = 0.33; *p* = 0.0053), and urinary fetuin-A was similarly correlated with dietary omega-3 intake (r = 0.26; *p* = 0.0241). In the control group, serum fetuin-A levels showed a negative correlation with MUFAs intake (r = −0.32; *p* = 0.0062), while urinary fetuin-A levels had a significant positive correlation with dietary protein intake (r = 0.29; *p* = 0.0172). However, there was no significant correlation between serum/urinary fetuin-A and the CDAI (*p* > 0.05).

## 4. Discussion

Identifying inhibitor factors that can prevent unwanted calcification, a major cause of kidney stone formation, is crucial for developing strategies to prevent both stone formation and recurrence. Research carried out for this purpose has gained momentum in recent years [6]. One of the potential inhibitory factors investigated is fetuin-A, which is one of the most powerful circulating inhibitors of hydroxyapatite formation [6,7].

Fetuin-A is suggested to have a high affinity for calcium ions, potentially inhibiting the creation and growth of hydroxyapatite crystals and preventing the deposition of calcium phosphate crystals [6,28]. In the studies by both Mehrsai et al. [6] and Arora et al. [12], which investigated serum and urine fetuin-A levels in kidney stone patients and healthy control groups, found that serum and urine fetuin-A levels were lower in the groups with kidney stones. Additionally, different studies have also shown that patients with kidney stones have lower urine fetuin-A levels [8,29]. Other findings from these studies indicate that high urinary calcium, oxalate, and serum creatinine levels, along with low urinary citrate levels, are also risk factors for kidney stone formation [6,8,12,29]. Nonetheless, other research in the literature indicates that there is no meaningful connection between nephrolithiasis and fetuin-A levels [13,20]. Differences in the methods of measuring fetuin-A and lack of standardization of study protocols may be the main reason for these different results. In this investigation, the kidney stone group’s serum and urine fetuin-A levels were considerably lower than those of the control group (*p* < 0.05). Similarly, serum potassium and urinary citrate levels were also lower in the kidney stone group (*p* < 0.05). In contrast, serum uric acid, creatinine, and urinary oxalate and creatinine levels were higher in the kidney stone group (*p* < 0.05) (Figure 2). Additionally, our findings revealed a substantial positive correlation between serum and urinary fetuin-A levels in the control group (*p* < 0.05). Another key finding was that in the control group, both serum and urinary fetuin-A levels exhibited a significant negative correlation with urinary oxalate and a positive correlation with urinary citrate (*p* < 0.05) (Figure 3). When the distribution of certain serum parameters was analyzed according to the quartiles of fetuin-A levels (serum and urine), urinary oxalate levels were lower in participants in the 2nd, 3rd, and 4th quartiles compared to those in the 1st quartile, while urinary citrate levels were higher. Furthermore, urinary citrate levels were higher in participants in the 3rd and 4th quartiles of serum fetuin-A compared to those in the 2nd quartile (*p* < 0.05) (Figure 3). Based on the data from both the literature and our study, it can be concluded that low serum and urinary fetuin-A levels may be a risk factor for nephrolithiasis by promoting crystallization and unwanted calcification. The relationships we found between fetuin-A and risk/inhibitory factors for stone formation support this hypothesis. Additionally, the present study reaffirms that low serum potassium and urinary citrate levels, along with high serum uric acid, creatinine, and urinary oxalate and creatinine levels, are common risk factors for kidney stone formation.

The results of the present study regarding serum and urinary fetuin-A are consistent with those of some similar studies [6,8,12], while they contradict the findings of others [13,20]. Possible confounding factors, such as differences in the kits used to measure serum and urinary fetuin-A levels, laboratory errors, the lack of a gold standard for measurement, fetuin-A gene polymorphisms, calcifying nanoparticles, and ethnic differences, may explain the inconsistencies between study results [6,8]. Additionally, it has been suggested that oxidative stress following kidney stone formation may gradually increase tubular damage and urinary calcium levels, leading to decreases in urinary fetuin-A levels, which could be one of the reasons for the observed inconsistencies in the results [6]. Therefore, to better understand the role of fetuin-A in kidney stone formation, prospective studies are needed that eliminate or standardize these confounding factors.

Identifying nutritional risk factors for nephrolithiasis may contribute significantly to the development of appropriate dietary interventions to prevent stone formation/recurrence and lessen the need for invasive surgeries for the treatment of the disease [14]. It is stated that the antioxidant/anti-inflammatory capacity of the diet is one of the factors affecting crystal nucleation/growth through its effects on intestinal flora, urine pH, and citrate levels [17]. The CDAI provides a comprehensive assessment of a diet’s capacity to reduce oxidative stress and neutralize free radicals [15], but its relationship to kidney stones is not yet clear [15,18]. Zhu et al. [15] found that higher CDAI levels were associated with reduced incidence and recurrence rates of kidney stones. Cui et al. [18], on the other hand, determined that both low and high CDAI scores were associated with a higher risk of developing kidney stones. In this study, total dietary energy (kcal/d), fiber (g), calcium (mg), phosphorus (mg) intakes, and the CDAI were higher in the kidney stone group than in the control group (*p* < 0.05). Given the limited number of studies investigating the nutritional risk factors for nephrolithiasis and the existence of contradictory results, it can be suggested that larger sample size prospective studies evaluating these relationships should be conducted. The risk of nephrolithiasis is said to be decreased by an increase in the CDAI up to a certain point, but the risk of stone formation may be increased by the CDAI above that point [18]. For example, taking more than the recommended dose of zinc, a component of the CDAI, can cause the development of nephrolithiasis by replacing calcium ions in crystals and promoting phosphate accumulation [18]. The results of the current study also support this hypothesis.

Determining the dietary factors that affect serum and urinary fetuin-A levels will help to more clearly elucidate the relationship between this biomarker and kidney stone development, as well as identify potential dietary variables that could modulate serum/urinary levels. It has been reported that serum/urinary fetuin-A levels are influenced by various dietary factors; some nutrients increase fetuin-A secretion, while others reduce it [19]. For example, Werida et al. [30] found that omega-3 supplementation of 1 g/day for 6 months increased plasma fetuin-A levels, while An et al. [31] observed that 3 g/day for 6 months also led to an increase in plasma fetuin-A levels. However, there are studies in the literature showing that omega-3 reduces serum fetuin-A levels [32] or is not significantly effective [33]. In the present study, serum fetuin-A was found to have a significant positive correlation with dietary monounsaturated fatty acids (MUFAs) intake (%) in the kidney stone group, while a significant negative correlation was observed in the control group (*p* < 0.05). When the potential relationships between urinary fetuin-A and dietary parameters were evaluated, a significant positive correlation was found between urinary fetuin-A and dietary protein (g) intake in the control group (*p* < 0.05). Another key finding of our study was the significant positive correlation between urinary fetuin-A and dietary omega-3 (g) intake in the kidney stone group (*p* < 0.05). Furthermore, participants in the 4th quartile of serum fetuin-A in the kidney stone group had higher dietary omega-3 intake than those in the 2nd and 3rd quartiles (*p* < 0.05). However, no significant correlation was found between serum/urinary fetuin-A and the CDAI (*p* > 0.05) (Figure 4). The data obtained suggest that some dietary parameters may affect serum/urine fetuin-A levels. However, the same effect cannot be said for the CDAI. This does not support the view that fetuin-A may be a marker of inflammatory-nutritional status [29].

There are some limitations of our study that should be taken into account. First, due to the cross-sectional nature of our study, it is not possible to prove causality. Stone formation is a multifactorial process, and the study groups were not matched for all possible stone causative factors. Another limitation is that ideal genetic matching was not possible. Although the average of three-day food records (consecutive days: two weekdays and one weekend day) was used to assess the CDAI, it may not reflect habitual diets and may contain bias. Another limitation is that the CDAI we used to assess the overall antioxidant capacity of participants’ diets does not account for all dietary antioxidants. In our study, the size of the kidney stones was not determined, and, therefore, the potential relationship between stone size and fetuin-A levels could not be examined. Additionally, the presence of other proteins, such as osteopontin [34], which were not included in our study but could have influenced the results and made individuals more prone to kidney stone formation, should also be considered.

However, our study has several strengths. To the best of our knowledge, this is the first study to investigate serum fetuin-A, urinary fetuin-A, oxalate, citrate levels, and some dietary parameters together with the CDAI in patients with kidney stones and healthy controls. In addition, our study has a larger sample size than all previous studies investigating the relationship between fetuin-A and nephrolithiasis [6,8,12,13,20,29,35], to our knowledge. Fetuin-A levels are strongly associated with age, gender, and BMI [36,37,38]. Therefore, the absence of significant differences between the groups in terms of age and BMI in the conducted studies is important for ensuring the reliability of the results. In the present study, the study groups were matched in terms of age, gender, and BMI. Thus, the results in our study were evaluated independently of this confounding factor. The findings of our study, conducted with a larger sample size compared to previous studies in the literature, may highlight the potential role of low serum and urinary fetuin-A levels in kidney stone development.

## 5. Conclusions

In conclusion, our findings suggest that fetuin-A levels in serum and urine are lower in kidney stone patients, which may prevent kidney stones from forming and may even be a predictive indicator of nephrolithiasis development on their own. Our hypothesis is supported by the finding that serum/urine fetuin-A levels have a positive correlation with certain recognized stone inhibitors (like urinary citrate) and a negative correlation with specific risk factors for stone formation (like urinary oxalate). An increase in the CDAI up to a certain level may reduce the risk of kidney stone formation, whereas the CDAI above a certain level may increase the risk of stone formation. Therefore, targeting the CDAI to be kept at certain levels may be a rational approach within kidney stone prevention strategies. Furthermore, it is expected that dietary intake of certain nutrients may influence serum/urinary fetuin-A levels. We hope that this study, which is the first to examine serum/urine fetuin-A levels, some biochemical parameters, anthropometric measurements, dietary parameters, and the CDAI in patients with kidney stones, will provide a basis for future research.

## Figures and Tables

**Table 1:** General characteristics of participants.

Characteristic	Kidney Stone Formers (n = 75)	Controls (n = 71)	*p*
Age (years)	42.1 ± 10.62	39.3 ± 11.16	0.053
Gender			
Male	51 (68.0)	39 (54.9)	0.105
Female	24 (32.0)	32 (45.1)	
Marital status			
Married or with partner	13 (17.3)	34 (47.9)	<0.001 *
Single	62 (82.7)	37 (52.1)	
Education level			0.005 *
≤High school	47 (62.7)	28 (39.4)	
>High school	28 (37.3)	43 (60.6)	
Smoking status			
Never smoker	63 (84.0)	52 (73.2)	0.112
Current smoker	12 (16.0)	19 (26.8)	
Alcohol consumption			
Yes	5 (6.7)	18 (25.4)	0.002 *
No	70 (93.3)	53 (74.6)	
Family history of stones			
Yes	30 (40.0)	9 (12.7)	<0.001 *
No	45 (60.0)	62 (87.3)	
Kidney stone recurrence			
1	13 (24.1)	-	-
2	15 (27.8)	-	
>2	26 (48.1)	-	
Number of main meals	2.6 ± 0.50	2.6 ± 0.49	0.693
Number of snacks	1.0 ± 0.83	1.3 ± 0.70	0.007 *
BMI (kg/m^2^)	27.5 ± 3.96	26.5 ± 4.28	0.608
BMI category			
Normal weight	21 (28.0)	28 (39.4)	
Overweight	33 (44.0)	30 (42.3)	
Obese	21 (28.0)	13 (18.3)	
Body fat percentage (%)	27.2 ± 7.95	28.5 ± 18.17	0.708
Body water percentage (%)	51.9 ± 5.66	52.4 ± 5.46	0.576
Body muscle mass (kg)	53.3 ± 8.61	53.3 ± 12.40	0.410
Waist circumference (cm)	99.5 ± 12.28	93.2 ± 15.93	0.001 *
Hip circumference (cm)	104.8 ± 10.29	104.8 ± 8.70	0.745
Waist/hip ratio	1.0 ± 0.72	0.9 ± 0.10	<0.001 *

* *p* < 0.05, Student’s *t*-test, Mann–Whitney U test, or chi-squared test. BMI, body mass index.

## Data Availability

The data presented in this study are available on request from the corresponding authors.

## Abbreviations

The following abbreviations are used in this manuscript:

CDAI	Composite dietary antioxidant index
ELISA	Enzyme-linked immunosorbent assay
FMC	Fetuin-mineral complex
CPP	Calciprotein particles
BMI	Body mass index
MUFAs	Monounsaturated fatty acids
SFAs	Saturated fatty acids
PUFAs	Polyunsaturated fatty acids
